# Computing dominant metric dimensions of certain connected networks

**DOI:** 10.1016/j.heliyon.2024.e25654

**Published:** 2024-02-06

**Authors:** Imtiaz Ali, Muhammad Javaid, Yilun Shang

**Affiliations:** a Department of Mathematics, University of Management and Technology, C-II, Johar Town, Lahore, Pakistan; b Department of Computer and Information Sciences, Northumbria University, Newcastle NE1 8ST, UK

**Keywords:** 05C12, 05C15, 05C78, Metric dimension, Dominant metric dimension, Connected networks

## Abstract

In the studies of the connected networks, metric dimension being a distance-based parameter got much more attention of the researches due to its wide range of applications in different areas of chemistry and computer science. At present its various types such as local metric dimension, mixed metric dimension, solid metric dimension, and dominant metric dimension have been used to solve the problems related to drug discoveries, embedding of biological sequence data, classification of chemical compounds, linear optimization, robot navigation, differentiating the interconnected networks, detecting network motifs, image processing, source localization and sensor networking. Dominant resolving sets are better than resolving sets because they carry the property of domination. In this paper, we obtain the dominant metric dimension of wheel, gear and anti-web wheel network in the form of integral numbers. We observe that the aforesaid networks have bounded dominant metric dimension as the order of the network increases. In particular, we also discuss the importance of the obtained results for the robot navigation networking.

## 1 Introduction

A network *G* contains a vertex set V(G) (nodes) and an edge set E(G) (lines) that link them [1]. In a network, *G*, the number of lines and vertices are called the size and order of the network respectively. A network *G* is connected if every two vertices are connected by a line. The distance between u,v∈V(G) is represented by d(u,v) as mentioned in [2]. An ordered set $R=\{v_{1},v_{2},v_{3},v_{4},v_{5},v_{6}\ldots ,v_{k}\}\subseteq V(G)$ is named as a resolving set if for ∀ a,b∈V(G), we have, r(a/R)≠r(b/R), where r(a/R) = $\left\{d(a,v_{1}),d(a,v_{2}),d(a,v_{3}\right)$, $d(a,v_{4}),\cdots ,d(a,v_{k})\}$. In [3], Charterend et al. talked about the applications of resolving sets in networks. A resolving set that carries minimum vertices is represented by *B* and is named the metric basis. The cardinality of a metric basis *B* which is represented by β(G) is named as the metric dimension of the network as mentioned in [4]. The expression x∼y means that *x* has an edge with *y* or simply xy∈E(G). A set S⊆V(G) is named as a dominating set if for each *y*∈ V(G)﹨S, there is at least one x∈S which satisfies x∼y. The dominating set among all the dominating sets of *G* which has the minimum cardinality is represented by *D*. The cardinality of the dominating set *D* which is denoted by γ(G) is named as the dominating number of *G* as mentioned in [5].

A dominating set which is also a resolving set is called a dominant resolving set as mentioned in [6]. A dominant resolving set that carries minimum vertices is named as a dominant metric basis of the network and is represented as $B_{d}$. The cardinality of $B_{d}$ (dominant metric basis) is named as the dominant metric dimension of the network and is represented by $\beta_{d}(G)$ as mentioned in [6]. Further, the authors in [6] obtained the $\beta_{d}(G)$ of complete bipartite $K_{m,n}$, cycle $C_{n}$, star $S_{n}$ and a path network $P_{n}$. For the study of strong metric dimension see [7], mixed metric dimension [8], edge metric dimension [9], local metric dimension [10], local fractional metric dimension [11] and fault-tolerant metric dimension [12].

Buczkowski et al. [13] worked out the expressions of the metric dimension of the wheel network. Since then the above-mentioned dimensions of wheel and related networks like sunflower, *t*-fold wheel, Helm, flower snarks, friendship, and convex polytopes have been studied by various authors. Javaid and Tomescu [14] obtained the expressions for the metric dimension of the gear network. Afzal et al. [15] worked out the metric dimension of the double wheel, *m*-level wheel, convex polytopes, and anti-web gear network. Ali et al. [36] formulated the expressions of the metric dimension of the Four-Dimensional Klein Bottle. Nadeem et al. [38] obtained the metric dimension of the generalized families of Toeplitz graphs. Imran et al. [16] determined the expressions of the metric dimension of the double gear, *m*-level gear, and generalized gear network. Naeem and Imran [17] studied the metric dimension of $AWW_{n}$. Afzal et al. [18] formulated the expressions for the metric dimension of the convex polytopes ($D_{n}$, $D_{n}^{2}$ and $D_{n}^{m}$).

Fault-tolerant metric dimension (FTMD) of gear and anti-web networks was computed by Liu et al. as mentioned in [19]. Zheng et al. obtained FTMD of convex polytopes and generalized wheels as mentioned in [20]. Imtiaz et al. [21] calculated the FTMD of multilevel wheel networks. Umilasari and Susilowati [22] studied the dominant local metric dimension of the wheel, Jahangir, and friendship network. Solekhah and Kusmayadi studied the local metric dimension of the generalized fan and *t*-fold wheel network in 2018 as mentioned in [23]. In [24], Javaid et al. formulated the local fractional metric dimension of the prism, m-level wheel, flower, anti-web gear, and Helm network. Javaid et al. [25] worked out the local fractional metric dimension of some families of generalized sun. In [37], [39], Saha et al. determined the FTMD of circulant graphs.

In this research, we obtained the dominant resolving sets and the dominant metric dimension of a gear, wheel, and anti-web wheel network.

## 2 Applications

There are various applications of the set of nodes in a network to remain identical at one or more than one location like the taxi trip deduction problem [40] and multi-agents deduction problem [41]. In particular, Tillquist et al. [26, Section 7], discussed the applications of metric dimension in source localization, detecting network motifs, and embedding biological sequence data problems. Laird et al. [27] obtained the metric dimension of hamming networks and discussed its uses in computational biology. Metric dimensions have been broadly used in robot navigation [28], telecommunication networks [30], graph theory [2], [29], chemistry [31], combinatorial optimization [32], sociology [33], as a basic set for fixing the order of DNA in real space [34] and geographical routing protocols [35]. For comprehensive details of resolving sets and metric dimensions, we refer the reader to [26].

## 3 Construction of networks

In this section, we defined some networks with figures. Further, we introduce some concepts which are helpful for this research paper.

**Figure 1:**
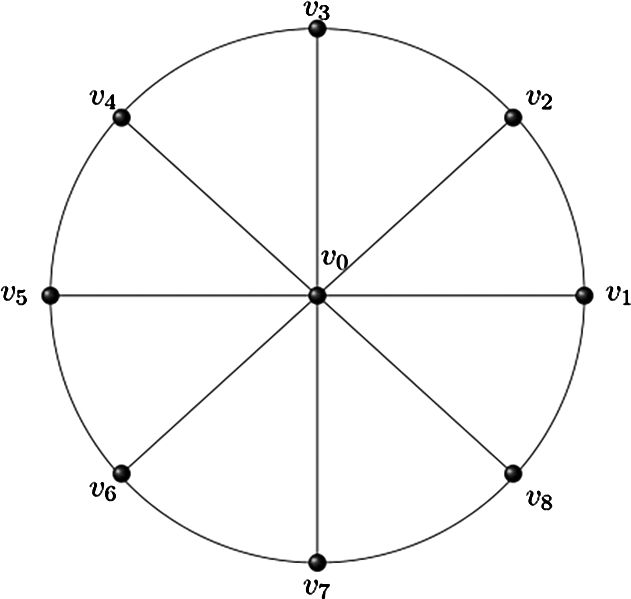
A network of the wheel (*W*_8_).

Definition 1 For a cycle $C_{n}$ and vertex $v_{0}$, we can obtain a wheel network by adding cycle $C_{n}$ to vertex $v_{0}$. Therefore $W_{n}\equiv v_{0}+C_{n}$. So $V(W_{n})=V(C_{n}\cup v_{0})$ while $E(W_{n})=E(C_{n}\cup v_{0}a:a\in V(C_{n}))$. Wheel network has order n+1 and size 2*n* (Fig. 1, [13]).

**Figure 2:**
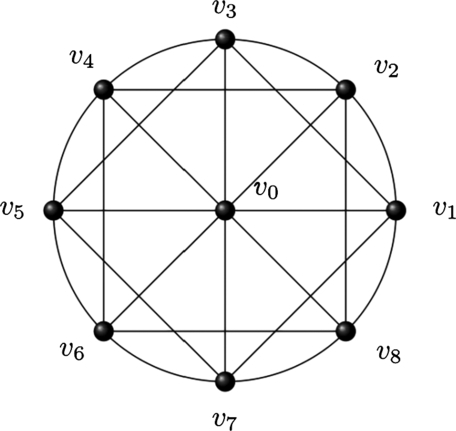
A network of *AWW*_8_.

Definition 2 For a wheel network $W_{n}$, if we add edges $\{v_{j}v_{j+2}$: $1\leq j\leq n,v_{j}\in C_{n}\}$ to wheel network $W_{n}$ then the new network obtained is named as anti-web wheel network $AWW_{n}$. Therefore, $V(W_{n})=V(AWW_{n})$ while $E(W_{n}\cup (v_{j}v_{j+2}$: $1\leq j\leq n,v_{j}\in C_{n}))=E(AWW_{n})$. Anti-web wheel network has order n+1 and size 3*n* (Fig. 2, [21]).

**Figure 3:**
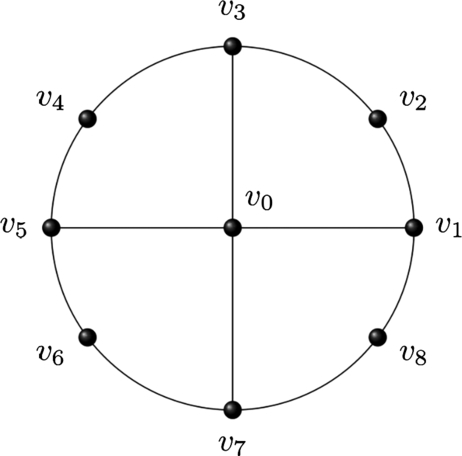
A network of *G*_8_.

Definition 3 For a wheel network, the gear network $G_{n}$ (where *n* is even) is obtained by deleting alternately *n* spokes from the wheel network. The order of $G_{n}$ is 2n+1 and its size is 3*n* (Fig. 3, [14]).

We categorize the vertices of wheel-related networks into two types. The vertices that belong to the dominant metric basis are represented by $B_{d}$ and the vertices that do not belong to the dominant metric basis are mentioned as a complement of the dominant metric basis and are represented by $B_{d}^{\prime }=V(G)﹨\;B_{d}$. Further, $B_{d}\cup B_{d}^{\prime }$ = V(G) and $B_{d}\cap B_{d}^{\prime }=\emptyset$. If V(G) = $\left\{v_{1},v_{2},v_{3},v_{4}...,v_{n}\right\}$ be the vertex set of a network *G* and its metric basis is *B* = $\left\{v_{i_{1}},v_{i_{2}},...,v_{i_{r}}\right\}$. Then the vertices $v_{i_{b}}$, $v_{i_{b+1}}$, where 1≤b≤r and $v_{i_{r+1}}$ = $v_{i_{1}}$ are mentioned as adjoining vertices in metric basis *B*. On a metric basis, the number of vertices between adjoining vertices is named as a gap of adjoining vertices. The cardinality of a gap is equal to the number of vertices in that gap. The Cardinalities of consecutive adjoining gaps are mentioned as $n_{1},n_{2},n_{3},n_{4},\ldots ,n_{r}$, where *r* is the number of gaps and *n* is a whole number. If the vertices {$v_{i_{b}}$, $v_{i_{b+1}}$, $v_{i_{b+2}}$, & $v_{i_{b+3}}$ } $\in B_{d}$, then the gaps in between {$v_{i_{b}}$, $v_{i_{b+1}}$}, {$v_{i_{b+1}}$, $v_{i_{b+2}}$} and {$v_{i_{b+2}}$, $v_{i_{b+3}}$} are referred to as left, central and right gaps respectively. Similarly, the gaps of dominant metric basis are mentioned as dominant gaps. In gaps and dominant gaps, we did not include vertex $v_{0}$.

In the gear network, (Fig. 3) we observed three types of vertices, the central vertex $v_{0}$, and the vertices with degrees 3 and 2. We represented the vertices with degrees 2 and 3 with even and odd numbering respectively. Further, we divided the gaps of the gear network into four types called odd-odd, odd-even, even-odd, and even-even gaps, where even and odd stand for vertices with degrees 2 and 3 respectively.

## 4 Main results

This section consists of three subsections in which we studied the dominant metric basis and dimension of wheel, gear, and anti-web wheel network. First, we obtained the dominant resolving sets of aforesaid networks by using the definition of the dominant resolving set then with the help of Lemmas we proved that dominant resolving sets have minimum cardinality. Finally, we computed the dominant metric dimensions.

Lemma 1 *In a connected network, if a set* W⊆V(G) *carries a resolving set then W is also a resolving set as mentioned in* *[6]* *.*

Lemma 2 *For a connected network G, if there is no dominating resolving set* R⊆V(G) *, where* |R|=n *, then there is also not a dominating resolving set* W⊂R⊆V(G) *, which satisfies* |W|<n *as mentioned in* *[6]* *.*

### 4.1 Dominant metric dimension of wheel network

Buczkowski et al. [13] while computing the metric dimension of the wheel network, observed five cases of minimum resolving sets which we included in Lemma 3. The minimum resolving sets obtained in [13] are also unique.

Lemma 3 *For unique and minimum resolving sets of*$W_{n}$*, the following five cases are observed as mentioned in**[13]**.*

1. *For*n≥10*,*n=5r*, where*r≥2*the resolving set B =*$\left\{v_{5j+1},v_{5j+4}:0\leq j\leq r-1\right\}$*is a metric basis.*
2. *For*n≥11*,*n=5r+1*, where*r≥2*, the resolving set B =*$\left\{v_{5j+1},v_{5j+4}:0\leq j\leq r-2\}\cup \{v_{5r-4},v_{5r}\right\}$*is a metric basis.*
3. *For*n≥12*,*n=5r+2*, where*r≥2*, the resolving set B =*$\left\{v_{5j+1},v_{5j+4}:0\leq j\leq r-1\}\cup \{v_{5r+1}\right\}$*is a metric basis.*
4. *For*n≥13*,*n=5r+3*, where*r≥2*, the resolving set B =*$\left\{v_{5j+1},v_{5j+4}:0\leq j\leq r-2\}\cup \{v_{5r-4},v_{5r},v_{5r+2}\right\}$*is a metric basis.*
5. *For*n≥14*,*n=5r+4*, where*r≥2*, the resolving set B =*$\left\{v_{5j+1},v_{5j+4}:0\leq j\leq r\right\}$*is a metric basis.*

Lemma 4 *There does not exist a gap in network*$W_{n}$*which contains 4 or more than 4 vertices.*

Proof We proved it by the contradiction method. The following cases are observed.

• Suppose there is a central gap in wheel network $W_{n}$ which carries 4 vertices and its left and right gaps have cardinality 0 (cardinalities in this case will be 0, 4, 0), then there will be vertices $v_{q},v_{q+1},v_{q+2},v_{q+3},v_{q+4},v_{q+5},v_{q+6}$, & $v_{q+7}$ $\in W_{n}$, where $v_{q},v_{q+1},v_{q+6}$, & $v_{q+7}\in B_{d}$, so there exists a contradiction as $r(v_{q+3}/B_{d})$ = $r(v_{q+4}/B_{d})$ = (2,2,2,2).
• Suppose there is a central gap in wheel network $W_{n}$ which carries 4 vertices and its left and right gaps have cardinality 0 and 1 (cardinalities in this case will be 0, 4, 1), then there will be vertices $v_{q},v_{q+1},v_{q+2},v_{q+3},v_{q+4},v_{q+5},v_{q+6}$, $v_{q+7}$, & $v_{q+8}$ $\in W_{n}$, where $v_{q},v_{q+1},v_{q+6}$, & $v_{q+8}\in B_{d}$, so there exists a contradiction as $r(v_{q+3}/B_{d})$ = $r(v_{q+4}/B_{d})$ = (2,2,2,2).
• Suppose there is a central gap in wheel network $W_{n}$ which carries 4 vertices and its left and right gaps have cardinality 0 and 1 (cardinalities in this case will be 1, 4, 1), then there will be vertices $v_{q},v_{q+1},v_{q+2},v_{q+3},v_{q+4},v_{q+5},v_{q+6}$, $v_{q+7},v_{q+8}$, & $v_{q+9}$ $\in W_{n}$, where $v_{q},v_{q+2},v_{q+7}$, & $v_{q+9}\in B_{d}$, so there exists a contradiction as $r(v_{q+4}/B_{d})$ = $r(v_{q+5}/B_{d})$ = (2,2,2,2). □

Theorem 1 *If*n≥10*, then*$\beta_{d}(W_{n})=\lfloor \frac{2n+4}{5}\rfloor$*.*

Proof We observed that, $\beta_{d}(W_{3})=3$ ($B_{d}$ = $\left\{v_{1},v_{2},v_{3}\right\}$), $\beta_{d}(W_{4})=2$ ($B_{d}$ = $\left\{v_{1},v_{2},\right\}$), $\beta_{d}(W_{5})=3$ ($B_{d}$ = $\left\{v_{1},v_{2},v_{3}\right\}$), $\beta_{d}(W_{6})=3$ ($B_{d}$ = $\left\{v_{1},v_{3},v_{5}\right\}$), $\beta_{d}(W_{7})=3$ ($B_{d}$ = $\left\{v_{1},v_{3},v_{5}\right\}$), $\beta_{d}(W_{8})=3$ ($B_{d}$ = $\left\{v_{0},v_{1},v_{3},v_{5}\right\}$) and $\beta_{d}(W_{9})=4$ ($B_{d}$ = $\left\{v_{1},v_{4},v_{6},v_{9}\right\}$). For n≥10, the following five cases are observed.

**Case 1:** If n≥10, n=5r, where r≥2, we choose $B_{d}$ = $\left\{v_{5j+1},v_{5j+4}:0\leq j\leq r-1\right\}$. Now by Lemma 3, $B_{d}$ contains a resolving set *B* = $\left\{v_{5j+1},v_{5j+4}:0\leq j\leq r-1\right\}$. So by Lemma 1, $B_{d}$ is also a resolving set.

Now

$$\left|B_{d}|=2r=\lfloor \frac{2n+4}{5}\right\rfloor$$

Or

$$\beta_{d}(W_{n})=\lfloor \frac{2n+4}{5}\rfloor .$$

**Case 2:** For n≥11, n=5r+1, where r≥2, we choose $B_{d}$ = $\left\{v_{5j+1},v_{5j+4}:0\leq j\leq r-2\}\cup \{v_{5r-4},v_{5r},v_{0}\right\}$. Now by Lemma 3, $B_{d}$ contains a resolving set *B* = $\left\{v_{5j+1},v_{5j+4}:0\leq j\leq r-2\}\cup \{v_{5r-4},v_{5r}\right\}$. So by Lemma 1, $B_{d}$ is also a resolving set.

Now

$$\left|B_{d}|=2r+1=\lfloor \frac{2n+4}{5}\right\rfloor$$

Or

$$\beta_{d}(W_{n})=\lfloor \frac{2n+4}{5}\rfloor .$$

**Case 3:** For n≥12, n=5r+2, where r≥2, we choose $B_{d}$ = $\left\{v_{5j+1},v_{5j+4}:0\leq j\leq r-1\}\cup \{v_{5r+1}\right\}$. Now by Lemma 3, $B_{d}$ contains a resolving set *B* = $\left\{v_{5j+1},v_{5j+4}:0\leq j\leq r-1\}\cup \{v_{5r+1}\right\}$. So by Lemma 1, $B_{d}$ is also a resolving set. Now

$$\left|B_{d}|=2r+1=\lfloor \frac{2n+4}{5}\right\rfloor$$

Or

$$\beta_{d}(W_{n})=\lfloor \frac{2n+4}{5}\rfloor .$$

**Case 4:** For n≥13, n=5r+3, where ≥2, we choose $B_{d}$ = $\left\{v_{5j+1},v_{5j+4}:0\leq j\leq r-2\}\cup \{v_{5r-4},v_{5r},v_{5r+2},v_{0}\right\}$. Now by Lemma 3, $B_{d}$ contains a resolving set *B* = $\left\{v_{5j+1},v_{5j+4}:0\leq j\leq r-2\}\cup \{v_{5r-4},v_{5r},v_{5r+2}\right\}$. So by Lemma 1, $B_{d}$ is also a resolving set.

Now

$$\left|B_{d}|=2r+2=\lfloor \frac{2n+4}{5}\right\rfloor$$

Or

$$\beta_{d}(W_{n})=\lfloor \frac{2n+4}{5}\rfloor .$$

**Case 5:** For n≥14, n=5r+4, where r≥2, we choose $B_{d}$ = $\left\{v_{5j+1},v_{5j+4}:0\leq j\leq r\right\}$. Now by Lemma 3, $B_{d}$ contains a resolving set *B* = $\left\{v_{5j+1},v_{5j+4}:0\leq j\leq r\right\}$. So by Lemma 1, $B_{d}$ is also a resolving set.

Now

$$\left|B_{d}|=2r+2=\lfloor \frac{2n+4}{5}\right\rfloor$$

Or

$$\beta_{d}(W_{n})=\lfloor \frac{2n+4}{5}\rfloor .$$

Now we show that $B_{d}$ is a dominating resolving set. We see that in cases 1, 3 and 5, $v_{1}∼v_{0}$, $v_{5j+1}∼v_{5j+2}$, $v_{5j+3}∼v_{5j+4}$ and $v_{5j+4}∼v_{5j+5}$. Similarly, in cases 2 and 4, we see that for each vertex $u\in V(W_{n})﹨B_{d}$ there is a $v_{0}\in B_{d}$, which satisfies $u∼v_{0}$. So for each $x\in V(W_{n})﹨B_{d}$ there is a $y\in B_{d}$, which satisfies x∼y. Therefore, $B_{d}$ is a dominating resolving set.

Now we show that the cardinality of $B_{d}$ is minimum. For this purpose, we apply Lemma 2. First, we prove it for cases 2 and 4. We observed the following cases.

1. Let $W\subseteq B_{d}$ does not carry $v_{0}$, now is a vertex $v_{5r-2}\in V(W_{n})﹨W$, such that for all v∈W, we obtain $v_{5r-2}≁v$, which is a contradiction.
2. Let $W\subseteq B_{d}$ does not carry one of the vertices of $B_{d}$ other than $v_{0}$. Now there exists a gap of cardinality 4. According to Lemma 4, in resolving sets of $W_{n}$, there can not exist a gap of cardinality 4 or greater than 4. So *W* is not a resolving set because it contains a gap of 4 vertices.

So in cases 2 and 4 $B_{d}$ satisfies Lemma 2. Therefore, in these cases the cardinality of $B_{d}$ is minimum.

Now we prove that the cardinality of $B_{d}$ is minimum for cases 1, 3, and 5. For this purpose, let $W\subseteq B_{d}$ not carry one of the vertices of $B_{d}$. Now there exists a gap of cardinality 4. According to Lemma 4, in resolving sets of $W_{n}$, there can not exist a gap of cardinality 4 or greater than 4. So in this case *W* is not a resolving set. Therefore, from the analysis of all the above cases, we concluded that the cardinality of $B_{d}$ is minimum.

Hence

$$\beta_{d}(W_{n})=\lfloor \frac{2n+4}{5}\rfloor .$$

 □

### 4.2 Dominant metric dimension of anti-web wheel network

We observed that in $AWW_{n}$ there are many gaps which have cardinality 3. The central vertex $v_{0}$ has edges with all the vertices of cycle $C_{n}$. For resolving sets we want gaps with greater cardinalities.

Lemma 5 *For*n≥8*, no gap of the resolving set of*$AWW_{n}$*contains 4 or more than 4 vertices.*

Proof We proved it by the contradiction method. The following cases are observed.

• Suppose there is a central gap in $AWW_{n}$ which carries 4 vertices and its left and right gaps are empty, (Cardinalities of these gaps are 0, 4, 0) then there will exist a metric basis *B* which contains vertices $v_{j},v_{j+1},v_{j+6}$, & $v_{j+7}$ in such a manner that $r(v_{j+5}/B)=(2,2,1,1,2,2,2,2,2)=r(v_{j+8}/B)$, a contradiction.
• Suppose there is a central gap in $AWW_{n}$ which carries 4 vertices and its left and right gaps contain 0 and 1 vertices respectively, (Cardinalities of these gaps are 0, 4, 1) then there will exit a metric basis *B* which contains vertices $v_{j},v_{j+1},v_{j+6}$, & $v_{j+8}$ in such a manner that $r(v_{j+4}/B)=(2,2,1,2,2,2,2,2,2)=r(v_{j+5}/B)$, a contradiction.
• Suppose there is a central gap in $AWW_{n}$ which carries 4 vertices and its left and right gaps contain 1 and 0 vertices respectively, (Cardinalities of these gaps are 1, 4, 0) then there will exist a metric basis *B* which contains vertices $v_{j},v_{j+2},v_{j+7}$, & $v_{j+8}$ such that $r(v_{j+3}/B)=(2,1,2,2,2,2,2,2)=r(v_{j+4}/B)$, a contradiction.
• Suppose there is a central gap in $AWW_{n}$ which carries 4 vertices and its left and right gaps contain 1 and 1 vertices respectively, (Cardinalities of these gaps are 1, 4, 1) then there will exit a metric basis *B* which contains vertices $v_{j},v_{j+2},v_{j+7}$, & $v_{j+9}$ such that $r(v_{j+3}/B)$ = $r(v_{j+4}/B)$ = (2, 1, 2, 2, 2, 2, 2), a contradiction.
• Suppose there is a central gap in $AWW_{n}$ which carries 4 vertices and its left and right gaps contain 2 and 1 vertices respectively, (Cardinalities of these gaps are 2, 4, 1) then there will exist a metric basis *B* which contains vertices $v_{j},v_{j+3},v_{j+8}$, & $v_{j+10}$ such that $r(v_{j+3}/B)=(2,1,2,2,2,2,2)=r(v_{j+4}/B)$, a contradiction. □

Lemma 6 *For*n≥8*, if a central gap of anti-web wheel network*$AWW_{n}$*of cardinality 3 exits then both of its left and right gaps can not have cardinality 2 or greater than 2.*

Proof The following cases are observed.

• Suppose there is a central gap in anti-web wheel network $AWW_{n}$ which carries 3 vertices and its left and right gaps contain 3 and 2 vertices respectively, (Cardinalities of these gaps are 3, 3, 2) then there will exist a metric basis *B* which contains vertices $v_{j},v_{j+4},vj+8$, & $v_{j+11}$ such that $r(v_{j+3}/B)=(2,1,2,2,2,2)=r(v_{j+5}/B)$, a contradiction.
• Suppose there is a central gap in anti-web wheel network $AWW_{n}$ which carries 3 vertices and its left and right gaps contain 2 and 3 vertices respectively, (Cardinalities of these gaps are 2, 3, 3) then there will exist a metric basis *B* which contains vertices $v_{j},v_{j+3},v_{j+7}$, & $v_{j+11}$ such that $r(v_{j+2}/B)$ = $r(v_{j+4}/B)$ = (2, 1, 2, 2, 2, 2), a contradiction.
• Suppose there is a central gap in anti-web wheel network $AWW_{n}$ which carries 3 vertices and its left and right gaps contain 2 and 2 vertices respectively, (Cardinalities of these gaps are 2, 3, 2) then there will exit a metric basis *B* which contains vertices $v_{j},v_{j+3},v_{j+7}$, & $v_{j+10}$ such that $r(v_{j+2}/B)=(2,1,2,2,2,2)=r(v_{j+4}/B)$, a contradiction.
• Suppose there is a central gap in anti-web wheel network $AWW_{n}$ which carries 3 vertices and its left and right gaps contain 2 and 1 vertices respectively, (Cardinalities of these gaps are 2, 3, 1) then there will exist a metric basis *B* which contains vertices $v_{j},v_{j+3},v_{j+7}$, & $v_{j+9}$ such that $r(v_{j+2}/B)=(2,1,2,2,2,\;2)=r(v_{j+4}/B)$, a contradiction.
• Suppose there is a central gap in anti-web wheel network $AWW_{n}$ which carries 3 vertices and its left and right gaps contain 1 and 2 vertices respectively, (Cardinalities of these gaps are 1, 3, 2) then there will exist a metric basis *B* which contains vertices $v_{j},v_{j+2},v_{j+6}$, & $v_{j+9}$ such that $r(v_{j+5}/B)=(2,2,1,2,2,2,2)=r(v_{j+7}/B)$, a contradiction. □

Lemma 7 *If*n=6r+2*and*r≥1*, then the set*$W=\{v_{6j+3},v_{6j+7}:0\leq j\leq r-1\}\cup \{v_{1}\}$*is a resolving set of*$AWW_{n}$*.*

Proof If n=6r+2 and r≥1, then the set $W=\{v_{6j+3},v_{6j+7}:0\leq j\leq r-1\}\cup \{v_{1}\}$ satisfies Lemma 5, Lemma 6. So it is a resolving set. □

Lemma 8 *If*n=6r+4*and*r≥1*, then the set*$W=\{v_{6j+3},v_{6j+7}:0\leq j\leq r-1\}\cup \{v_{1},v_{n-1}\}$*is a resolving set.*

Proof If n=6r+4 and r≥1, then the set $W=\{v_{6j+3},v_{6j+7}:0\leq j\leq r-1\}\cup \{v_{1},v_{n-1}\}$ satisfies Lemma 5, Lemma 6. So it is a resolving set. □

Lemma 9 *If*n=6r*and*r≥2*, then the set*$W=\{v_{6j+3},v_{6j+7}:0\leq j\leq r-1\}\cup \{v_{1},v_{n-3}\}$*is a resolving set.*

Proof If n=6r and r≥2, then the set $W=\{v_{6j+3},v_{6j+7}:0\leq j\leq r-1\}\cup \{v_{1},v_{n-3}\}$ satisfies Lemma 5, Lemma 6. So it is a resolving set. □

Theorem 2 *If* n≥8 *, then* $\beta_{d}(AWW_{n})=\lfloor \frac{n+5}{3}\rfloor$ *.*

Proof We observed that, $\beta_{d}(AWW_{4})=4$, where $B_{d}$ = $\left\{v_{1},v_{2},v_{3},v_{4}\right\}$ and $\beta_{d}$ $\left(AWW_{6}\right)$ =3, where $B_{d}$ = $\left\{v_{1},v_{3},v_{5}\right\}$. For n≥8, the following three cases are observed.

**Case 1:** If r≥1 and n=6r+2, then we choose $B_{d}=\{v_{6j+3},v_{6j+7}:0\leq j\leq r-1\}\cup \{v_{0},v_{1}\}$. By Lemma 7, $W=\{v_{6j+3},v_{6j+7}:0\leq j\leq r-1\}\cup \{v_{1}\}$ is a resolving set. AS $B_{d}$ contains *B*. So it satisfies Lemma 1.

Now

$$\left|B_{d}|=2r+2=\lfloor \frac{n+5}{3}\right\rfloor$$

Or

$$\beta_{d}(AWW_{n})=\lfloor \frac{n+5}{3}\rfloor .$$

**Case 2:** If n=6r+4 and r≥1, then we choose $B_{d}=\{v_{6j+3},v_{6j+7}:0\leq j\leq r-1\}\cup \{v_{0},v_{1},v_{n-1}\}$. By Lemma 8, $B=\{v_{6j+3},v_{6j+7}:0\leq j\leq r-1\}\cup \{v_{1},v_{n-1}\}$ is a resolving set. AS $B_{d}$ contains *B*. So it satisfies Lemma 1.

Now

$$\left|B_{d}|=2r+3=\lfloor \frac{n+5}{3}\right\rfloor$$

Or

$$\beta_{d}(AWW_{n})=\lfloor \frac{n+5}{3}\rfloor .$$

**Case 3:** If n=6r and r≥2, then we choose $B_{d}=\{v_{6j+3},v_{6j+7}:0\leq j\leq r-1\}\cup \{v_{0},v_{1},v_{n-3}\}$. By Lemma 9, $B_{d}=\{v_{6j+3},v_{6j+7}:0\leq j\leq r-1\}\cup \{v_{0},v_{1},v_{n-3}\}$ is a resolving set. AS $B_{d}$ contains *B*. So it satisfies Lemma 1.

Now

$$\left|B_{d}|=2r+1=\lfloor \frac{n+5}{3}\right\rfloor$$

Or

$$\beta_{d}(AWW_{n})=\lfloor \frac{n+5}{3}\rfloor .$$

Now we show that $B_{d}$ is a dominating resolving set. We see that for each vertex $v\in V(AWW_{n})﹨B_{d}$ there is a vertex $v_{0}\in B_{d}$, which satisfies $v_{0}∼v$. Therefore, $B_{d}$ is a dominating resolving set.

Now in order to show that the cardinality of $B_{d}$ is minimum, we observe the following two cases.

1. Let $W\subseteq B_{d}$ does not carry $v_{0}$, now there is a vertex $v_{6j+5}\in V(AWW_{n})﹨W$, such that for all v∈W, we obtain $v_{5j+3}≁v$, which is a contradiction.
2. Let $W\subseteq B_{d}$ does not carry one of the vertices other than $v_{0}$. Now there exists a gap of cardinality 4. According to Lemma 5, in resolving sets of $AWW_{n}$, there can not exist a gap of cardinality 4 or greater than 4. Therefore, *W* is not a resolving set as it does not satisfy Lemma 5.

So $B_{d}$ satisfies Lemma 2. Therefore, cardinality of $B_{d}$ is minimum.

Hence

$$\beta_{d}(AWW_{n})=\lfloor \frac{n+5}{3}\rfloor .$$

 □

### 4.3 Dominant metric dimension of gear network

In this part, we obtained the dominant metric dimension of the gear network. We observed from [18] that in gear network $G_{n}$ (in [18] it is represented as $J_{2n}$), there are many gaps which have cardinality 4 and the central vertex $v_{0}$ did not have edges with the vertices which have degree 2. So a dominant gap can not contain four vertices. We detected the following five Lemmas for the gear network.

Lemma 10 *There does not exist a dominant gap of 4 vertices in gear network*$G_{n}$*.*

Proof As in gear network $G_{n}$ even-even or odd-odd gaps can contain only an odd number of vertices so it is clear that there do not exist even-even or odd-odd gaps of 4 vertices. Now suppose there exists an even-odd gap of 4 vertices, then there exists vertices $v_{i},v_{i+1},v_{i+2}$, & $v_{i+3}$ belong to $B_{d}^{\prime }$ = $V(G_{n})﹨B_{d}$ such that there is a vertex $v_{i+1}$ with degree 2 which has no edge with any $v\in B_{d}$. Similarly, if there exists odd-even gap of 4 vertices, then their exists vertices $v_{i},v_{i+1},v_{i+2}$, & $v_{i+3}$ belong to $B_{d}^{\prime }=V(G_{n})﹨B_{d}$ such that there is a vertex $v_{i+2}$ with degree 2 which has no edge with any $v\in B_{d}$. □

Lemma 11 *There does not exist an even-even dominant gap of 3 vertices in gear network*$G_{n}$*.*

Proof Suppose there exists an even-even gap of 3 vertices in gear network $G_{n}$, then there exist vertices $v_{i},v_{i+1}$, & $v_{i+2}$ belong to $B_{d}^{\prime }=V(G_{n})﹨B_{d}$ such that there is a vertex $v_{i+1}$ with degree 2 which has no edge with any $v\in B_{d}$, a contradiction. □

Lemma 12 *If an odd-odd gap of 3 vertices in gear network*$G_{n}$*exists in a resolving set, then its left and right gaps can contain 1 and 1 vertex respectively.*

Proof We proved it by contradiction method by making different cases.

• Suppose there is an odd-odd gap in gear network $G_{n}$ which carries 3 vertices and its left and right gaps also contain 3 and 3 vertices respectively, (Cardinalities of these gaps are 3, 3, 3) then there will exist a resolving set *W* which contains vertices $v_{j},v_{j+4},v_{j+8}$, & $v_{j+12}$ such that $r(v_{j+2}/B)=(2,2,2,2)=r(v_{j+6}/B)$, a contradiction.
• Suppose there is an odd-odd gap in gear network $G_{n}$ which carries 3 vertices and its left and right gaps contain 3 and 2 vertices respectively, (Cardinalities of these gaps are 3, 3, 2) then there will exist a resolving set *W* which contains vertices $v_{j},v_{j+4},v_{j+8}$, & $v_{j+11}$ such that $r(v_{j+2}/B)=(2,2,2,2)=r(v_{j+6}/B)$, a contradiction.
• Suppose there is an odd-odd gap in gear network $G_{n}$ which carries 3 vertices and its left and right gaps contain 2 and 3 vertices respectively, (Cardinalities of these gaps are 2, 3, 3) then there will exist a resolving *W* which contains vertices $v_{j},v_{j+3},v_{j+7}$, & $v_{j+11}$ such that $r(v_{j+6}/B)=(3,2,2,2)=r(v_{j+10}/B)$, a contradiction.
• Suppose there is an odd-odd gap in gear network $G_{n}$ which carries 3 vertices and its left and right gaps also contain 2 and 2 vertices respectively, (Cardinalities of these gaps are 2, 3, 2) then there will exist a resolving set *W* which contains vertices $v_{j},v_{j+3},v_{j+7}$, & $v_{j+10}$ such that $r(v_{j}/B)=(1,2,2,3)=r(v_{j+2}/B)$, a contradiction.
• Suppose there is an odd-odd gap in gear network $G_{n}$ which carries 3 vertices and its left and right gaps also contain 2 and 1 vertices respectively, (Cardinalities of these gaps are 2, 3, 1) then there will exist a resolving set *W* which contains vertices $v_{j},v_{j+3},v_{j+7}$, & $v_{j+9}$ such that $r(v_{j+2}/B)=(1,2,2,2)=r(v_{j+6}/B)$, a contradiction.
• Suppose there is an odd-odd gap in gear network $G_{n}$ which carries 3 vertices and its left and right gaps also contain 1 and 2 vertices respectively, (Cardinalities of these gaps are 1, 3, 2) then there will exit a resolving set *W* which contains vertices $v_{j},v_{j+2},v_{j+6}$, & $v_{j+9}$ such that $r(v_{j+2}/B)=(2,2,2,1)=r(v_{j+6}/B)$, a contradiction. □

Lemma 13 *At most one an odd-odd gap of cardinality 3 can exist in gear network*$G_{n}$*in a resolving set.*

Proof Since from Lemma 12, if an odd-odd gap exists in the gear network then its left or right gaps can not contain 3 vertices. Now we suppose that in a resolving set, a central gap contains 1 vertex and its left and right gaps contain 3 and 3 vertices respectively. So there exists vertices in $v_{i},v_{i+4},v_{i+6}$, & $v_{i+9}$ such that $r(v_{i+2}/B)=(2,2,2,2)=r(v_{i+8}/B)$, a contradiction.

So it follows from the above Lemmas if we include a gap with 3 vertices in the resolving set which is only one, then we have to choose a dominant resolving set which contains $v_{0}$. So we will not include a gap of 3 vertices in resolving sets because there can exist at most one gap with 3 vertices. □

Lemma 14 *There do not exist two consecutive odd-even and even-odd gaps in the dominant resolving set of gear network*$G_{n}$*which contain 2 and 2 vertices respectively.*

Proof Suppose that there exist two consecutive odd-even and even-odd gaps in gear network $G_{n}$ which contain 2 and 2 vertices respectively, then there will exit vertices $v_{i},v_{i+2}$, & $v_{i+4}$ in resolving set such that $r(v_{i}/B)=(1,2,3)=r(v_{i+2}/B)$), a contradiction. □

Theorem 3 *If*n≥10*, then*$\beta_{d}(G_{n})=\lceil \frac{3n}{8}\rceil$*.*

Proof We observes that, $\beta_{d}(G_{4})=3$, $\beta_{d}(G_{6})=3$ and $\beta_{d}(G_{8})=3$, where $B_{d}$ = $\left\{v_{2},v_{3},v_{4}\right\}$, $B_{d}$ = $\left\{v_{1},v_{3},v_{5}\right\}$ and $B_{d}$ = $\left\{v_{2},v_{5},v_{8}\right\}$, are their dominant metric basis respectively. For n≥10, the following four cases are observed.

**Case 1:** For n=8r+2, where r≥1, the set $B_{d}$ = $\left\{v_{8j+5},v_{8j+8},v_{8j+10}:0\leq j\leq r-1\}\cup \{v_{2}\right\}$ is a resolving set because it satisfies Lemma 10, Lemma 14.

Now

$$\left|B_{d}|=3r+1=\lceil \frac{3n}{8}\right\rceil$$

Or

$$\beta_{d}(G_{n})=\lceil \frac{3n}{8}\rceil .$$

**Case 2:** For n=8r+4, where r≥1, the set $B_{d}$ = $\left\{v_{8j+5},v_{8j+8},v_{8j+10}:0\leq j\leq r-1\}\cup \{v_{2},v_{n}\right\}$ is a set because it satisfies Lemma 10, Lemma 14.

Now

$$\left|B_{d}|=3r+2=\lceil \frac{3n}{8}\right\rceil$$

Or

$$\beta_{d}(G_{n})=\lceil \frac{3n}{8}\rceil .$$

**Case 3:** For n=8r+6, where r≥1, the set $B_{d}$ = $\left\{v_{8j+5},v_{8j+8},v_{8j+10}:0\leq j\leq r-1\}\cup \{v_{2},v_{n-1},v_{n}\right\}$ is a set because it satisfies Lemma 10, Lemma 14.

Now

$$\left|B_{d}|=3r+3=\lceil \frac{3n}{8}\right\rceil$$

Or

$$\beta_{d}(G_{n})=\lceil \frac{3n}{8}\rceil .$$

**Case 4:** For n=8r+8, where r≥1, the set $B_{d}$ = $\left\{v_{8j+5},v_{8j+8},v_{8j+10}:0\leq j\leq r-1\}\cup \{v_{2},v_{n-3},v_{n}\right\}$ is a set because it satisfies Lemma 10, Lemma 14.

Now

$$\left|B_{d}|=3r+3=\lceil \frac{3n}{8}\right\rceil$$

Or

$$\beta_{d}(G_{n})=\lceil \frac{3n}{8}\rceil .$$

Now we show that $B_{d}$ is a dominating resolving set. Here, $v_{1}∼v_{2}$, $v_{3}∼v_{2}$, $v_{1}∼v_{0}$, $v_{8j+4}∼v_{8j+5}$, $v_{8j+6}∼v_{8j+5}$, $v_{8j+7}∼v_{8j+8}$, $v_{8j+9}∼v_{8j+10}$, $v_{8j+10}∼v_{8j+11}$ and $v_{n-1}∼v_{n}$. We see that for each vertex $u\in V(G_{n})﹨B_{d}$ there is a vertex $v\in B_{d}$, which satisfies u∼v. Therefore, $B_{d}$ is a dominating resolving set.

Now in order to show that the cardinality of $B_{d}$ is minimum. Let $W\subseteq B_{d}$ does not carry one of the vertices of $B_{d}$. Now there exists a gap of cardinality 4. According to Lemma 10, in dominant resolving sets of $G_{n}$, there can not exist a gap of cardinality 4 or greater than 4. So *W* is not a dominant resolving set. Therefore $B_{d}$ satisfies Lemma 2. So cardinality of $B_{d}$ is minimum.

Hence

$$\beta_{d}(G_{n})=\lceil \frac{3n}{8}\rceil .$$

 □

## 5 Discussion

Buczkowski et al. [23], Tomescu et al. [24] and Imran et al. [27] obtained the metric dimensions of the wheel, gear, and anti-web wheel network respectively as follows.

1. $\beta (W_{n})=\lfloor \frac{2n+2}{5}\rfloor$
2. $\beta (J_{2n})=\lfloor \frac{2n}{3}\rfloor$
3. $\beta (AWW_{n})=\lfloor \frac{n+2}{3}\rfloor$.

Liu et al. [29], Zheng et al. [30] and Imtiaz et al. [31] obtained the fault-tolerant metric dimension denoted by $\beta^{\prime }(G)$ of the wheel, gear, and anti-web wheel network respectively as follows.

1. $\beta^{\prime }(W_{n})=\lceil \frac{n}{2}\rceil$
2. $\beta^{\prime }(G_{n})=\frac{n}{2}$
3. $\beta^{\prime }(AWW_{n})=\frac{n}{2}$.

In this paper, we obtained the following results.

1. $\beta_{d}(W_{n})=\lfloor \frac{2n+4}{5}\rfloor$
2. $\beta_{d}(AWW_{n})=\lfloor \frac{n+5}{3}\rfloor$
3. $\beta_{d}(G_{n})=\lceil \frac{3n}{8}\rceil$.

If we compare the above-mentioned results, then we obtained the following inequalities.

1. $\beta (W_{n})\leq \beta_{d}(W_{n})\leq \beta^{\prime }(W_{n})$
2. $\beta (AWW_{n})\leq \beta_{d}(AWW_{n})\leq \beta^{\prime }(AWW_{n})$
3. $\beta (G_{n})\leq \beta_{d}(G_{n})\leq \beta^{\prime }(G_{n})$.

Now if robots are navigating on a wheel network, where vertices of wheels are landmarks and edges are paths on which robots move, then this research helps decision-makers who want to minimize the number of robots a navigating on wheel network and to find the location of these robots in such a manner that distances to the landmarks uniquely control robot position on wheel network. Various types of wheel networks are explained and their metric dimensions are formulated. Decision makers can choose a wheel network according to the situation and can apply the formula to obtain the minimum number of robots for navigation on the network. Similarly, if we consider a wheel graph as a wheel network for communications, then metric dimension and resolving sets of wheel-related networks are useful in such wheel network communications.

## 6 Conclusion

In this research, we studied the dominant metric dimension of the wheel, gear, and anti-web wheel network. The two networks anti-web wheel and gear network are obtained by adding and deleting edges from the wheel network respectively. In this research, we checked variations in the dominant metric dimension by varying the number of edges of a wheel network. We first obtained the dominant resolving sets of aforesaid networks and then computed the dominant metric dimension of these networks. We observed that if we add edges in the wheel network to form the anti-web wheel network then the dominant metric dimension decreases and if we delete edges from the wheel network to form a gear network then again dominant metric dimension decreases. Consequently, in this study, the dominant metric dimension of the wheel network decreases by adding or deleting edges in the wheel network. So if we consider the vertices of a wheel network or wheel-related network as computers and edges as wires and the purpose is to find a network that has a less dominant metric dimension with less number of edges then the gear network is suitable. If the decision maker wants to find a network with less dominant metric dimension and more edges then the anti-web wheel network is suitable among the three wheel, gear, and anti-web wheel network.

## Disclosure statement

We declare that Yilun Shang is a Section Editor of Heliyon.

## Funding statement

The work of Muhammad Javaid was supported by the 10.13039/501100010221Higher Education Commission of Pakistan through the National Research Program for Universities under Grant 20-16188/NRPU/R&D/HEC/2021.

## Additional information

No additional information is available for this paper.

## CRediT authorship contribution statement

**Imtiaz Ali:** Writing – review & editing, Writing – original draft, Investigation, Formal analysis, Conceptualization. **Muhammad Javaid:** Writing – review & editing, Writing – original draft, Investigation, Formal analysis, Conceptualization. **Yilun Shang:** Writing – review & editing, Writing – original draft, Investigation, Formal analysis, Conceptualization.

## Declaration of Competing Interest

The authors declare that they have no known competing financial interests or personal relationships that could have appeared to influence the work reported in this paper.

## Data Availability

No data was used for the research described in the article.
